# Comparative Evaluation of Two Bracket Systems’ Kinetic Friction: Conventional and Self-Ligating

**DOI:** 10.3390/ma15124304

**Published:** 2022-06-17

**Authors:** Aurel-Claudiu Vartolomei, Dan-Cosmin Serbanoiu, Dana-Valentina Ghiga, Marioara Moldovan, Stanca Cuc, Maria Cristina Figueiredo Pollmann, Mariana Pacurar

**Affiliations:** 1 Faculty of Dental Medicine, GEP University of Medicine Pharmacy, Science and Technology of Targu Mures, 540139 Târgu Mureș, Romania; claudiu.vartolomei@gmail.com (A.-C.V.); serbanoiu.dancosmin@gmail.com (D.-C.S.); valentinaghiga@gmail.com (D.-V.G.); marianapac@yahoo.com (M.P.); 2 Raluca Ripan Chemistry Research Institute, Babes Bolyai University, 400294 Cluj-Napoca, Romania; marioara.moldovan@ubbcluj.ro (M.M.); stanca.boboia@ubbcluj.ro (S.C.); 3 Faculty of Dental Medicine, University of Porto, 4200-393 Porto, Portugal

**Keywords:** friction, self-ligating brackets, conventional brackets, tensile strength, maximum load

## Abstract

Friction is an intensely studied feature in orthodontics, as the sliding mechanics approach remains one of the most utilized techniques in current practice, and the question of whether self-ligating brackets produce less friction than conventional brackets still stands. The objective of this study was to compare a self-ligating system with different closing mechanisms and a conventional system with different ligating mechanisms regarding their frictional properties. Laboratory measurements were performed using an advanced materials testing machine generating tensile strength and load at maximum Load values, which were statistically analyzed and compared. These two parameters have been associated with the frictional resistance generated at the archwire–bracket slot interface. Statistically significant results were obtained when comparing the active self-ligating brackets with the passive self-ligating (tensile strength mean 1.953, SD 0.4231; load at maximum moad mean 6.000, SD 1.3000) and conventional brackets (tensile strength mean 1.953, SD 0.4231; load at maximum load mean 6.000, SD 1.3000), as well as when comparing the passive self-ligating brackets with the conventional brackets (tensile strength mean 1.708, SD 0.8628; load at maximum load mean 5.254, SD 2.645). The active self-ligating brackets tended to produce more friction when compared to the passive self-ligating brackets but were similar to conventional brackets with stainless steel ligatures.

## 1. Introduction

One of the essential aspects of orthodontic brackets is the ligating system, as it maintains the archwire engaged in the bracket slot, permitting dental movement, although it also represents one of the main friction-generating factors. Stainless steel and elastomeric ligatures have been mainly utilized in conventional bracket systems. The steel ligatures’ dimensions range from 0.009” to 0.012” in diameter. They maintain their shape and do not corrode in the oral environment [1]. Elastomeric ligatures were introduced in the 1970s. They fail to fully engage the archwire in the slot and have been shown to increase friction when applying sliding mechanics. They also undergo force decay and permanent deformation [2,3].

Self-ligating brackets do not use wire or elastomeric ligatures to engage the archwire into the bracket slot, as they present their own built-in locking mechanism. There are two types of self-ligating brackets, depending on the design of the mechanism: active, whereby the locking mechanism consists of a resilient but flexible clip that actively engages the archwire into the bracket slot once it reaches a certain size or deflection; and passive, whereby the slot is locked by a rigid locking mechanism, basically turning the bracket into a tube, allowing the archwire to slide freely within it [4].

There is constant debate on the frictional characteristics of the self-ligating and conventional bracket systems. Manufacturers claim that self-ligating braces produce less friction in comparison with conventional braces [5]. Friction represents “The function of the relative roughness of two interacting surfaces and results when the two relative surfaces move against each other” [6]. There are two basic types of friction: static and kinetic. Static friction is “The lowest force required to initiate orthodontic tooth movement when two surfaces are statically related”, while kinetic friction equals “The force that resists the movement of one object against another when a constant speed is applied” [7,8]. Static and kinetic friction occur if the wire is in a passive position, parallel to the bracket, although this is seldom encountered in clinical settings [9]. Tooth movement occurs when an applied force overcomes the friction at the bracket slot–archwire contact point. However, in order to preserve anchorage and avoid a high risk of root resorption and increased levels of pain, low forces are be preferred [10]. Thus, it is important to acknowledge the friction between the bracket and archwire in order to apply proper force levels to obtain adequate tooth movement and an optimum biological response [11].

The novelty of this study consists of bonding the brackets on the teeth and placing them in the testing machine, the method in which the archwire is pulled (and not the bracket along the wire), and the comparative combinations of bracket systems and ligating methods, including the load at maximum load parameter. Previous studies have placed multiple brackets on a metal bar, as the archwire moved at a crosshead speed of 0.5 mm [12], or have performed experiments in Chatillon, TCD 200, CSC Force Measurement, and Agawam mass testing machines [13]. Other research has focused on testing incisor brackets by sliding the archwire through multiple braces [14]. Similar research [15] involved sliding straight sections of archwire through canine brackets. This study also has the advantage of testing curved wires, reflecting the intraoral treatment, whereby the sliding mechanics approach do not apply to perfectly straight sections.

The purpose of this study is to compare the frictional characteristics of a self-ligating and a conventional bracket system using laboratory measurements.

## 2. Materials and Methods

A total of 90 stainless steel upper first premolar brackets (*n* = 90) bonded on 90 extracted teeth were utilized in the study as follows: 30 conventional metal brackets (15 tied with elastomeric ligatures and 15 with stainless steel ligatures), 30 active self-ligating brackets, and 30 passive self-ligating brackets. The used prescription for both systems involved ROTH 0.022-inch slot Master Series conventional brackets, Empower 2 Interactive (active) self-ligating brackets and passive self-ligating brackets from American Orthodontics. The archwire was 0.019/0.025 inch stainless steel, Europe I form. This is the most frequent archwire size used in sliding mechanics in the 0.022 inch slot when friction is at its peak due to larger teeth movements. The extracted teeth were acquired from several dental clinics in Romania. These were examined and only the intact ones with at least two-thirds of the root or roots and proper anatomical buccal surfaces suitable for bonding premolar braces were included. Teeth with cavities, fillings, or scales on the bonding surfaces were excluded from the start.

**Figure 1:**
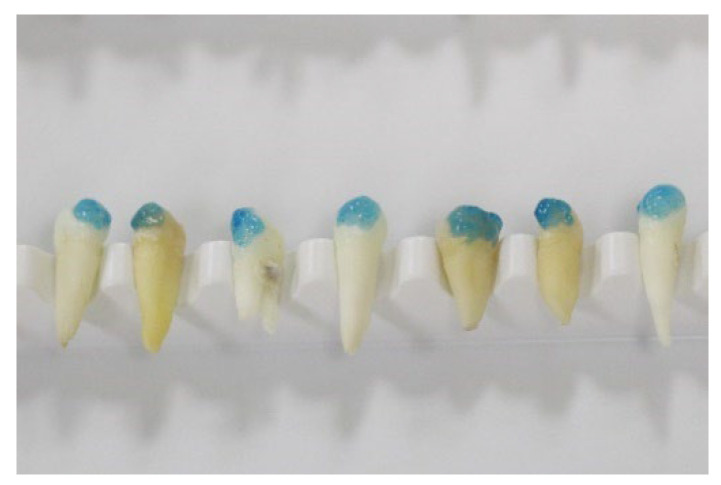
Etched premolars.

**Figure 2:**
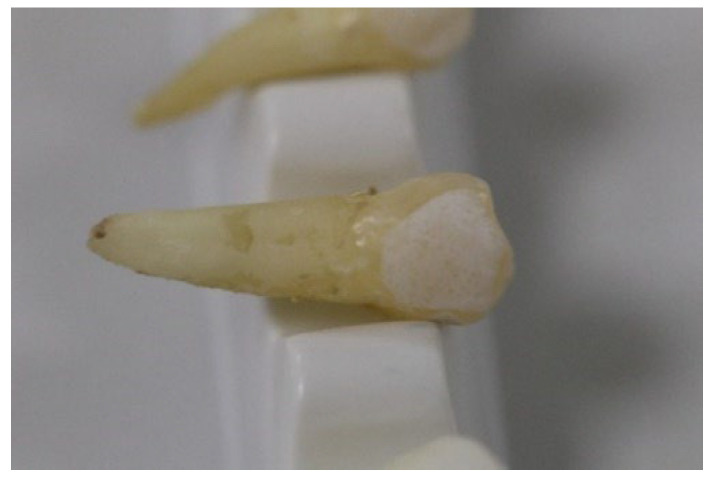
Etched premolars.

The teeth were cleaned with saline solution and conserved in artificial saliva immediately after extraction. The artificial saliva comprised disodium phosphate, sodium bicarbonate, calcium chloride, hydrochloric acid, and water. The percentages are presented in Table 1. The teeth were brushed, etched (orthophosphoric acid 36%, Blue Etch (Cerkamed)) for 20 s (as seen in Figure 1 and Figure 2), rinsed, and dried. Ormco Orthosolo Universal Bond Enhancer was applied and lightly cured with a Woodpecker iLed instrument (3 s), the braces were bonded with 3 M Transbond XT, and the excess composite was removed. The teeth were further preserved in artificial saliva for 1 month until the measurements were performed [16,17].

Resistance to friction in the brackets was analyzed by means of an ASTM (American Society for Testing and Materials) D638 compression test with an Lloyd LR5K Plus dual-column advanced materials testing machine (Ametek/Lloyd Instruments, Meerbusch, Germany), with a maximum capacity of 5 kN. The machine has an electronic compression force transmission and measurement system. Data processing was performed using NexygenPlus software.

For reproducibility, the following basic settings were selected for the tests: direction: tension, preload/stress: 50 N; preload/stress speed: 25 mm/min; autozero—automatically zero at the start of the test; load and extension, test speed—extension rate: 100 mm/min.

The teeth with the brackets and the ligated archwires were firmly positioned in the load cell, which was placed in the machine in a fixed reproducible position, so that the archwire would be pulled as close as possible to 0 degrees angulation on the first sector until its curvature. The upper vise pulled the archwire upwards with the specified parameters, simulating the orthodontic sliding mechanics.

**Figure 3:**
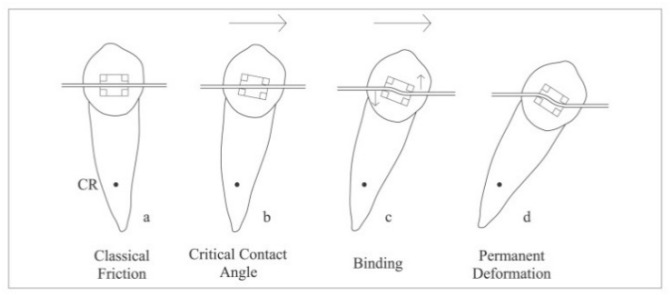
Friction, binding, and notching [22].

**Figure 4:**
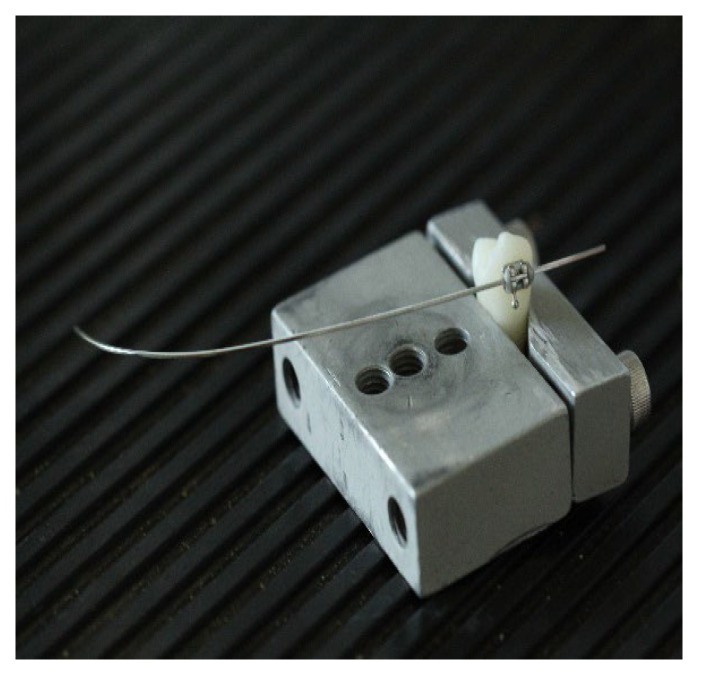
Locked premolar conventional bracket with elastomeric ligature.

**Figure 5:**
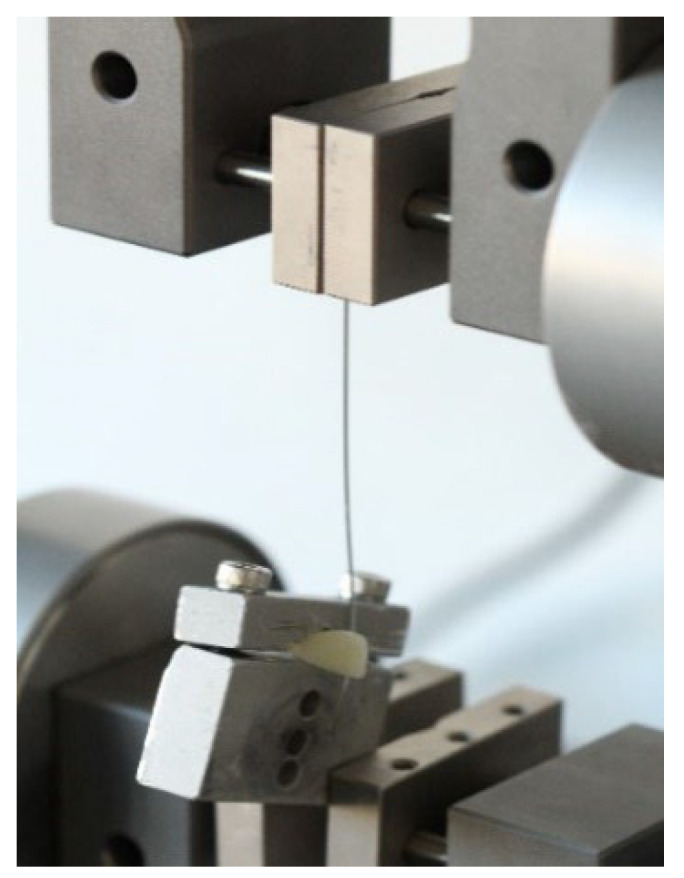
Half archwire and upper vise pulling the archwire.

If there is angulation between the archwire and the bracket slot and the wire comes in contact with its edges (in a critical contact angle for binding), binding starts to interfere [18]. The archwire is elastically deformed during the binding phenomenon, and due to its elasticity it tends to return to its previous original shape, generating binding of the wire at the opposing corners of the bracket slot [19,20]. At higher angulations, the archwire can no longer tolerate the forces of the slot walls and it begins to permanently deform. This is called notching, and it results in little or no movement of the tooth [21]. Friction, binding, and notching are graphically represented in Figure 3. The test stages can be found in Figure 4 and Figure 5.

The tensile strength is the maximum stress that a material can tolerate before breakage while being pulled or stretched [23]. The tensile strength can be quantified by performing a tensile test and recording the stress–strain curve. The highest point of this curve is the tensile strength [24]. It is measured as the force per unit area. In the International System of Units, the unit is the pascal (Pa) (or megapascals (MPa) newton per square meter (N/m2). [25]. The maximum load is the limit load that a structure can carry. It is measured in N in physics, and it is the load at which the structure is in a state of incipient plastic collapse.

The computer displayed the stress–strain graph followed by the tensile strength and load at the maximum load parameters. Their values were proportional and were correlated with the friction at the bracket slot–archwire interface, as the only force to be exerted was friction between the components. The values recorded corresponded to the point with the highest level of frictional force. Thirty-five measurements for the active self-ligating brackets (ASLB), 34 measurements for the passive self-ligating brackets (PSLB), 29 measurements for the conventional brackets tied with elastomeric ligatures (CBEL), and 24 measurements for the conventional brackets tied with stainless steel ligatures (CBSSL) were performed. The values were then compared, as can be seen in Table 2 (left column versus right column), and are presented in the same order in the Results section of the article. Scanning electron microscopy images of the tested archwires were taken after friction measurements for each group in order to verify the surfaces for subtle notching phenomena.

The statistical analysis comprised descriptive statistics elements (mean, median, standard deviation) and inferential statistics elements. The Shapiro–Wilk test was applied in order to determine the distribution of the analyzed data series. The distribution was non-parametric. The t-Student’s test was applied for a means comparison and the Mann–Whitney test was applied for a medians comparison. The chosen significance level for the *p*-value was 0.05. The statistical analysis was performed in the demo GraphPad Prism utility program.

## 3. Results

Dunn’s multiple comparison post hoc test was performed (the results can be seen in Table 3 and Table 4).

The following two tables (Table 5 and Table 6) comprise the means and standard deviations of all comparison groups for the tensile strength and load at maximum load values, along with the *p*-values. The detailed statistical tables can be accessed in the article’s Appendix A section. Nevertheless, the tables with all performed measurements are available on request.

ASLB-PSLB: Regarding the tensile strength values, the median values in active self-ligating brackets were significantly higher than the median values in passive self-ligating brackets (*p* < 0.05), and similar results were obtained when the load at maximum load values were compared (*p* < 0.05). This equals more friction in active self-ligating brackets.

**Figure 6:**
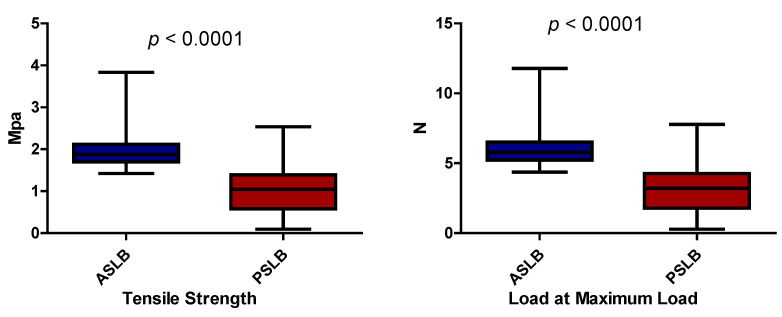
Maximum and minimum measured values for active and passive self-ligating brackets.

Maximum and minimum values and median intervals for the active and passive self-ligating brackets can be seen for the tensile strength and load at maximum load parameters (with a statistically significant difference) in Figure 6.

**ASLB-CB**: There was a statistically significant difference (*p* < 0.05) between the medians of the tensile strength values for the active self-ligating and conventional brackets. The median values were higher for the active self-ligating brackets. The load at maximum load median values were statistically higher as well (*p* < 0.05). The conventional brackets group comprised conventional brackets ligated with both elastomeric and stainless steel ligatures. This meant higher friction in the active self-ligating system.

**PSLB-CB**: Both the tensile strength and load at maximum load median values were significantly higher in conventional brackets when compared to passive self-ligating brackets (*p* < 0.05). This corresponded to higher friction in the conventional brackets.

**CBEL-CBSSL**: The medians of the tensile strength values were statistically higher in conventional brackets with stainless steel ligatures in comparison to conventional brackets with elastic ligatures (*p* < 0.05). The same result was obtained for the load at maximum load medians (*p* < 0.05). This translated into more friction in conventional brackets with stainless steel ligatures.

**ASLB-CBSSL**: The median tensile strength values for the active self-ligating brackets were lower compared to the median values for conventional brackets with stainless steel ligatures but were not statistically significant (*p* > 0.05). The same result was obtained for the load at maximum load values (*p* > 0.05). This meant similar frictional levels in the active self-ligating brackets and conventional brackets with stainless steel ligatures.

**PSLB-CBSSL**: Concerning the tensile strength values, the median values in conventional brackets with stainless steel ligatures were significantly higher than the median values in passive self-ligating brackets (*p* < 0.05). Similar statistical results were obtained when the load at maximum load values were compared (*p* < 0.05). This corresponded to less friction in passive self-ligating brackets.

## 4. Discussion

When comparing the active self-ligating brackets with the other systems, the obtained tensile strength and load at maximum load values were, in every case, higher for the ASLB. This transposes clinically to the highest friction levels at the bracket slot–archwire interface for this system. Contrariwise, the lowest forces of friction were generated in the passive self-ligating system. Similar studies have arrived to similar conclusions [26,27,28].

In our study, comparable values were found for the active self-ligating and conventional brackets with stainless steel ligatures. Thorstenson et al. found in their study that opened self-ligating brackets are similar to conventional stainless steel brackets concerning the resistance to sliding when both are ligated with stainless steel ligatures with the same amount of force [29]. This suggests that the closing mechanism is important when taking into consideration the friction reduction and supports our study’s comparisons. Additionally, in the case of stainless steel ligatures, the amount of ligation force should be taken into consideration. Naturally, strongly tied ligatures exert more friction. However, loose ligatures will not engage the archwire sufficiently in the slot and bracket information will not be clinically fully expressed.

During tooth movement, static and kinetic friction are the results of the archwire contact with the bracket and the ligating system [8]. This resulting friction contributes to a small part of the entire resistance to sliding during tooth movement. Friction is, thus, correlated with many aspects, including the type of bracket [30,31], sizes of the wire and alloy [32,33], ligation method [34,35], angulation between the bracket slot and archwire [36], and biological factors [37]. The biological factors include the saliva quality, bone density, position of the tooth in the medullary bone, occlusal interferences, soft tissue thickness, and growth pattern.

Other studies have assessed the different archwire–bracket–angle combinations and archwire materials and concluded that friction proportionally increases with bracket–wire angulation, and that in conventional brackets stainless steel archwires have the lowest friction, while in self-ligating brackets nickel–titanium archwires have the lowest friction values [10]. Our study focused on stiff, heavy stainless steel archwires, as these are the main working phase archwires when sliding mechanics takes place.

A similar test was conducted in wet conditions. The tree types of self-ligating brackets displayed lower frictional forces when compared to conventional brackets, and the passive self-ligating brackets exerted less friction than the active self-ligating brackets in these conditions [15].

The most recent view on this matter supports the belief that resistance to bodily tooth movement by sliding has little to do with friction, and instead is largely a binding-and-release phenomenon that is about the same with conventional and self-ligating brackets [19].

**Figure 7:**
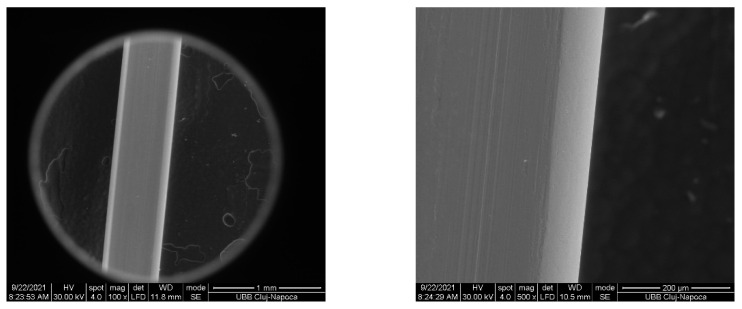
SEM image of the archwire after the test, with magnification at 100× and 500×.

The values measured for each test were obtained at the maximum curvature of the archwire, where resistance to sliding is at its highest. In contrast with the clinical situation, the generated friction did not prevent the movement of the archwire, as the strength of the testing machine overcame it. However, no permanent deformation of the archwires occurred during the tests. This can be noticed in the scanning electronic microscope images (Figure 7).

In the case of the conventional bracket system, the elastomeric ties generated less friction than the stainless steel ligatures. It is stated in the literature that elastomeric ties tend to increase friction [2,3]. Other aspects to be taken into consideration are the force degradation of the elements used to apply the sliding mechanics (power chain) and the force degradation caused by tooth movement (coil spring, laceback ligatures) in the intraoral environment [38,39]. In our study, the movement generated by the testing machine was constant (25 mm/min), with minimum interference from external factors. Reducing friction is of paramount importance in orthodontics in order to obtain faster teeth movement and reduce patient discomfort. Different strategies are currently being developed and studied. Coating nickel–titanium archwires with ZnO nanoparticles reduced the friction by up to 21% and also reduced the bacterial activity [40]. A new category of elastomeric ligatures (Metafasix), which are coated with a water-resistant polymer, making them very slippery in the presence of saliva, has been reported to decrease friction by 60% [41]. A polyurethane elastomeric ligature called Slide is another low-friction material that has been recently introduced into the market. Combined with a conventional bracket, this ligature forms a tube-like structure, and significantly lower friction forces were reported in comparison with conventional ligatures [42]. A diamond-like carbon surface coating of stainless steel and nickel-titanium archwires has been suggested to reduce frictional forces [43]. These ions increased the wire stiffness and reduced friction when compared to conventional wires. The technology was also tested to improve the clinical performance of stainless steel brackets [44]. The choice of bracket material also influences the friction. Stainless steel brackets produce less friction than polycrystalline alumina, which is why metal brackets are preferred to aesthetic ones [45].

The findings of this study are to be considered in light of the limitations associated with in vitro studies. This linear experiment cannot mimic the dynamic interactions that occur in the three-dimensional intraoral environment. This study was designed to observe classical friction at the bracket slot–archwire interface by discarding as many variables as possible. Moreover, the operation of the testing machine was performed manually, which can lead to some measurement errors. The lack of homogeneity of the methodologies applied in multiple studies has resulted in certain reservations regarding the comparative analysis of the results.

## 5. Conclusions

The most relevant outcome of this study is that active self-ligating brackets generated the greatest amounts of friction, closely followed by the conventional system with stainless steel ligatures, while the passive self-ligating brackets exerted the lowest friction forces.

## Figures and Tables

**Table 1:** Artificial saliva components.

Composition	%
Na_2_HPO_4_	0.3
NaHCO_3_	
CaC_l2_	
HCl-1M	0.3
H_2_O	99.4

**Table 2:** Brackets to be compared.

ASLB	PSLB
ASLB	CB
PSLB	CB
CBEL	CBSSL
ASLB	CBSSL
PSLB	CBSSL

**Table 3:** Dunn’s post hoc test—tensile strength.

Dunn’s Multiple Comparison Test	Significant? *p* < 0.05?
ASLB vs. PSLB	<0.0001
ASLB vs. CB	0.0034
ASLB vs. CBEL	<0.0001
ASLB vs. CBSSL	0.4826
PSLB vs. CB	0.0004
PSLB vs. CBEL	0.0994
PSLB vs. CBSSL	<0.0001
CB vs. CBEL	0.1127
CB vs. CBSSL	0.0721
CBE vs. CBSSL	0.0035

**Table 4:** Dunn’s post hoc test—load at maximum load.

Dunn’s Multiple Comparison Test	Significant? *p* < 0.05?
ASLB vs. PSLB	<0.0001
ASLB vs. CB	0.0033
ASLB vs. CBEL	<0.0001
ASLB vs. CBSSL	0.4730
PSLB vs. CB	0.0004
PSLB vs. CBEL	0.0938
PSLB vs. CBSSL	<0.0001
CB vs. CBEL	0.1150
CB vs. CBSSL	0.0739
CBEL vs. CBSSL	0.0037

**Table 5:** Means and SD—tensile strength.

Mean ± SD (Median)	ASLB (*n* = 35)	CB (*n* = 53)	PSLB (*n* = 34)	CBEL (*n* = 29)	CBSSL (*n* = 24)
Tensile Strength	1.953 ± 0.4231 (1.882)	1.708 ± 0.8628 (1.390)	1.030 ± 0.5301 (1.045)	1.505 ± 0.8938 (1.170)	1.954 ± 0.7714 (1.826)
*p*-values	ASLB vs.	* 0.0034	* < 0.0001		* 0.4826
	PSLB vs.	* 0.0004			<0.0001
	CBEL vs.				* 0.0035

* Mann–Whitney test.

**Table 6:** Means and SD—load at maximum load.

Mean ± SD (Median)	ASLB (*n* = 35)	CB (*n* = 53)	PSLB (*n* = 34)	CBEL (*n* = 29)	CBSSL (*n* = 24)
Load at Maximum Load	6.000 ± 1.300 (5.780)	5.254 ± 2.645 (4.270)	3.165 ± 1.629 (3.210)	4.633 ± 2.737 (3.594)	6.004 ± 2.370 (5.611)
*p*-values	ASLB vs.	* 0.0033	* <0.0001		* 0.4730
	PSLB vs.	* 0.0004			<0.0001
	CBEL vs.				* 0.0037

* Mann–Whitney test.

## Data Availability

Not applicable.

## Appendix A

**Table A1:** Tensile strength: active vs. passive self-ligating brackets.

Tensile Strength	ASLB	PSLB
Number of values	35	34
Minimum	1.423	0.09287
25% Percentile	1.699	0.5815
Median	1.882	1.045
75% Percentile	2.120	1.390
Maximum	3.835	2.535
Mean	1.953	1.030
Std. Deviation	0.4231	0.5301
Std. Error	0.07151	0.09092
Table Analyzed		
Column A		ASLB
vs.		vs.
Column B		PSLB
Mann Whitney test		
*p* value		*p* < 0.0001
Are medians signif. different? (*p* < 0.05)		Yes

**Table A2:** Load at maximum load: active vs. passive self-ligating brackets.

Load at Maximum Load	ASLB	PSLB
Number of values	35	34
Minimum	4.370	0.2853
25% Percentile	5.220	1.786
Median	5.780	3.210
75% Percentile	6.510	4.269
Maximum	11.78	7.787
Mean	6.000	3.165
Std. Deviation	1.300	1.629
Std. Error	0.2197	0.2793
Lower 95% CI of mean	5.554	2.597
Upper 95% CI of mean	6.447	3.733
Table Analyzed		
Column A		ASLB
vs.		vs.
Column B		PSLB
Mann Whitney test		
*p* value		*p* < 0.0001
Are medians signif. different? (*p* < 0.05)		Yes

**Table A3:** Tensile strength: active vs. conventional stainless steel ligature brackets.

Tensile Strength	ASLB	CBSSL
Number of values	35	24
Minimum	1.423	0.7672
25% Percentile	1.699	1.393
Median	1.882	1.826
75% Percentile	2.120	2.449
Maximum	3.835	4.030
Mean	1.953	1.954
Std. Deviation	0.4231	0.7714
Std. Error	0.07151	0.1575
Table Analyzed		
Column A		ASLB
vs.		vs.
Column B		CBSSL
Mann Whitney test		
*p* value		0.4730
Are medians signif. different? (*p* < 0.05)		No

**Table A4:** Load at maximum load: active vs. conventional stainless steel ligature brackets.

Load at Maximum Load	ASLB	CBSSL
Number of values	35	24
Minimum	4.370	2.357
25% Percentile	5.220	4.278
Median	5.780	5.611
75% Percentile	6.510	7.523
Maximum	11.78	12.38
Mean	6.000	6.004
Std. Deviation	1.300	2.370
Std. Error	0.2197	0.4838
Lower 95% CI of mean	5.554	5.003
Upper 95% CI of mean	6.447	7.004
Table Analyzed		
Column A		ASLB
vs.		vs.
Column B		CBSSL
Mann Whitney test		
*p* value		0.4730
Are medians signif. different? (*p* < 0.05)		No

**Table A5:** Tensile strength: active self-ligating vs. conventional brackets.

Tensile Strength	ASLB	CB
Number of values	35	53
Minimum	1.423	0.7045
25% Percentile	1.699	1.069
Median	1.882	1.390
75% Percentile	2.120	2.253
Maximum	3.835	4.030
Mean	1.953	1.708
Std. Deviation	0.4231	0.8628
Std. Error	0.07151	0.1185
Table Analyzed		
Column A	ASLB	
vs.	vs.	
Column B	CB	
Mann Whitney test		
*p* value	0.0034	
Exact or approximate *p* value?	Gaussian Approximation	
*p* value summary	**	
Are medians signif. different? (*p* < 0.05)	Yes	

The asterisks indicate how many zeros come after point in *p* values.

**Table A6:** Load at maximum load: active self-ligating vs. conventional brackets.

Load at Maximum Load	ASLB	CB
Number of values	35	53
Minimum	4.370	2.243
25% Percentile	5.220	3.283
Median	5.780	4.270
75% Percentile	6.510	6.922
Maximum	11.78	12.38
Mean	6.000	5.254
Std. Deviation	1.300	2.645
Std. Error	0.2197	0.3633
Table Analyzed		
Column A	ASLB	
vs.	vs.	
Column B	CB	
Mann Whitney test		
*p* value	0.0033	
Exact or approximate *p* value?	Gaussian Approximation	
*p* value summary	**	
Are medians signif. different? (*p* < 0.05)	Yes	

The asterisks indicate how many zeros come after point in *p* values.

**Table A7:** Tensile strength: passive self-ligating vs. conventional brackets.

Tensile Strength	PSLB	CB
Number of values	34	53
Minimum	0.09287	0.7045
25% Percentile	0.5815	1.069
Median	1.045	1.390
75% Percentile	1.390	2.253
Maximum	2.535	4.030
Mean	1.030	1.708
Std. Deviation	0.5301	0.8628
Std. Error	0.09092	0.1185
Table Analyzed		
Column A	PSLB	
vs.	vs.	
Column B	CB	
Mann Whitney test		
*p* value	0.0004	
Exact or approximate *p* value?	Gaussian Approximation	
*p* value summary	***	
Are medians signif. different? (*p* < 0.05)	Yes	

The asterisks indicate how many zeros come after point in *p* values.

**Table A8:** Load at maximum load: passive self-ligating vs. conventional brackets.

Load at Maximum Load	PSLB	CB
Number of values	34	53
Minimum	0.2853	2.243
25% Percentile	1.786	3.283
Median	3.210	4.270
75% Percentile	4.269	6.922
Maximum	7.787	12.38
Mean	3.165	5.254
Std. Deviation	1.629	2.645
Std. Error	0.2793	0.3633
Table Analyzed		
Column A	PSLB	
vs.	vs.	
Column B	CB	
Mann Whitney test		
*p* value	0.0004	
Exact or approximate *p* value?	Gaussian Approximation	
*p* value summary	***	
Are medians signif. different? (*p* < 0.05)	Yes	

The asterisks indicate how many zeros come after point in *p* values.

**Table A9:** Tensile strength: conventional system elastic ligatures vs. stainless steel ligatures.

Tensile Strength	CBEL	CBSSL
Number of values	29	24
Minimum	0.7045	0.7672
25% Percentile	0.9399	1.393
Median	1.170	1.826
75% Percentile	1.587	2.449
Maximum	3.615	4.030
Mean	1.505	1.954
Std. Deviation	0.8938	0.7714
Std. Error	0.1660	0.1575
Table Analyzed		
Column A	CBEL	
vs.	vs.	
Column B	CBSSL	
Mann Whitney test		
*p* value	0.0035	
Exact or approximate *p* value?	Gaussian Approximation	
*p* value summary	**	
Are medians signif. different? (*p* < 0.05)	Yes	

The asterisks indicate how many zeros come after point in *p* values.

**Table A10:** Load at maximum load: conventional system elastic ligatures vs. stainless steel ligatures.

Load at Maximum Load	CBEL	CBSSL
Number of values	29	24
Minimum	2.243	2.357
25% Percentile	2.906	4.278
Median	3.594	5.611
75% Percentile	4.875	7.523
Maximum	11.11	12.38
Mean	4.633	6.004
Std. Deviation	2.737	2.370
Std. Error	0.5083	0.4838
Table Analyzed		
Column A	CBEL	
vs.	vs.	
Column B	CBSSL	
Mann Whitney test		
*p* value	0.0037	
Exact or approximate *p* value?	Gaussian Approximation	
*p* value summary	**	
Are medians signif. different? (*p* < 0.05)	Yes	

The asterisks indicate how many zeros come after point in *p* values.

**Table A11:** Tensile strength: passive self-ligating vs. conventional brackets with stainless steel ligatures.

Tensile Strength	PSLB	CBSSL
Number of values	34	24
Minimum	0.09287	0.7672
25% Percentile	0.5815	1.393
Median	1.045	1.826
75% Percentile	1.390	2.449
Maximum	2.535	4.030
Mean	1.030	1.954
Std. Deviation	0.5301	0.7714
Std. Error	0.09092	0.1575
Table Analyzed		
Column A	PSLB	
vs.	vs.	
Column B	CBSSL	
Unpaired t test with Welch’s correction		
*p* value	*p* < 0.0001	
*p* value summary	***	
Are means signif. different? (*p* < 0.05)	Yes	

The asterisks indicate how many zeros come after point in *p* values.

**Table A12:** Load at maximum load: passive self-ligating vs. conventional with stainless steel ligatures.

Load at Maximum Load	PSLB	CBSSL
Number of values	34	24
Minimum	0.2853	2.357
25% Percentile	1.786	4.278
Median	3.210	5.611
75% Percentile	4.269	7.523
Maximum	7.787	12.38
Mean	3.165	6.004
Std. Deviation	1.629	2.370
Std. Error	0.2793	0.4838
Table Analyzed		
Column A	PSLB	
vs.	vs.	
Column B	CBSSL	
Unpaired t test with Welch’s correction		
*p* value	*p* < 0.0001	
*p* value summary	***	
Are means signif. different? (*p* < 0.05)	Yes	

The asterisks indicate how many zeros come after point in *p* values.
